# Engineering *Salmonella* as intracellular factory for effective killing of tumour cells

**DOI:** 10.1038/srep30591

**Published:** 2016-07-28

**Authors:** Eva María Camacho, Beatriz Mesa-Pereira, Carlos Medina, Amando Flores, Eduardo Santero

**Affiliations:** 1 Centro Andaluz de Biología del Desarrollo/CSIC/Universidad Pablo de Olavide/Junta de Andalucía. Departamento de Biología Molecular e Ingeniería Bioquímica, Seville, Spain

## Abstract

Salmonella have many desirable properties as antitumour-agent due to its ability to proliferate inside tumours and induce tumour regression. Additionally, this bacterium can be genetically engineered to deliver therapeutic proteins intratumourally. The main limitation of this approach is the efficient release of therapeutic molecules from intratumoural bacteria. Here we have developed an inducible autolysis system based in the lysis operon of the lambda phage that, in response to anhydrotetracycline, lysates Salmonella thus releasing its content. The system was combined with a salicylate cascade system that allows efficient production of therapeutic molecules in response to aspirin and with a *sifA* mutation that liberates bacteria from the vacuoles to a cytosolic location. The combination of these three elements makes this strain a putative powerful instrument in cancer treatment. We have used this engineered strain for the intracellular production and delivery of Cp53 peptide. The engineered strain is able to sequentially produce and release the cytotoxic peptide while proliferating inside tumour cells, thus inducing host cell death. Our results show that temporal separation of protein production from protein release is essential to efficiently kill tumour cells. The combined system is a further step in the engineering of more efficient bacteria for cancer therapy.

Living bacteria have been used as therapeutics agents since the end of the 19th century (reviewed in^1^) because they offer several advantages compared to therapeutic proteins such as being easier to grow, store and purify. This approach can be used in the treatment of illnesses naturally refractory to conventional drugs such as tumours^2^,^3^,^4^. *Salmonella enterica* serovar Typhimurium (*S. typhimurium*) is probably the intracellular pathogen that has been most extensively studied as an anti-tumour vector due to its intrinsic properties. These bacteria preferentially colonise and proliferate in solid tumours at ratios greater than 1000/1 compared to normal target organs, a behaviour that usually results in tumour growth inhibition^5^. In addition, as a facultative anaerobe, *Salmonella* can grow under either aerobic or anaerobic conditions, which allows bacteria to accumulate in large solid tumours (including hypoxic area) and invade metastases^6^,^7^.

Modifying bacteria for the intratumoural production of therapeutic molecules^1^,^2^,^4^ can increase the natural antitumour effectiveness of some bacteria. These molecules can include cytotoxic proteins, cytokines, tumour-specific antigens and antibodies, small harping RNAs, genes in eukaryotic vectors for bactofection, etc. The intracellular localisation of bacteria such as *Salmonella* allows the cytosolic delivery of drugs as iRNA or some cytotoxic proteins that are unable to enter eukaryotic cells. For these systems, the regulation of gene expression is crucial to control the timing and localisation of drug production to maximise the therapeutic effect while minimising any potential adverse effects over normal tissues. Several gene expression systems have been developed to trigger drug production in response to suitable non-toxic signals^2^,^8^.

The use of *Salmonella* as an effective intracellular factory also requires efficient cytosolic delivery systems that allow the drug export into the cytosol. *Salmonella* are facultative intracellular bacteria that are found within a variety of phagocytic and non-phagocytic cells *in vivo*^9^. Following internalisation, *Salmonella* survives and replicates within a modified vacuole known as the *Salmonella*-containing vacuole (SCV). Thus, to deliver the antitumour molecule into de cytosol, the therapeutic drug produced must be able to cross both, the bacterial envelope and the SCV membrane. For some treatments, such as those based in pro-drug converting enzyme or bacterial toxins, this is not a further constraint^1^,^10^,^11^,^12^. However, in the majority of cases, therapeutic molecules to be released from intracellular bacteria are not able to cross both, the bacterial envelope and the SCV. Fusions of therapeutic peptides to secretion signals have been used to facilitate crossing the bacterial envelope^12^,^13^,^14^,^15^,^16^ or both bacterial envelope and SCV^17^,^18^,^19^,^20^,^21^. However, this approach is useless when peptide activity is compromised by the presence of the secretion signal or peptides are not efficiently secreted. In addition, it is not applicable for bactofection (releasing of eukaryotic expression vectors) or treatment with shRNA. In bactofection delivery of therapeutics genes usually depends on spontaneous release from bacteria and SCV^22^,^23^. For shRNA treatment, peptides such as listeriolysin are produced by bacteria to allow passage of this RNA through the membrane of the vacuole^24^,^25^,^26^,^27^ but still drug release depends on spontaneous bacterial lysis. In spite of these obvious limitations, most treatments have yielded promising results.

An alternative method that surpasses all these limitations is the use of programed lysis of bacteria. Lysis systems have been used to release heteromacromolecules from bacteria to the growth medium^28^,^29^. Recently, a lysis system based on lysis genes of a newly characterised *Salmonella* phage have been developed to release heteromacromolecules from intratumoural *Salmonella typhimurium*^30^. In spite of promising results obtained, this system has some limitations: (i) Bacteria still remain enclosed by the SCV; therefore, the tested antitumoural proteins need to be fused to a cell-penetrating peptide to allow their delivery into the cytosol, thus limiting the type of heteromolecules produced; (ii) both, the production of antitumour protein and the lysis system are coupled to the P_*bad*_ promoter, limiting antitumour protein production to the time elapsed between induction and lysis; (iii) the expression system shows evident basal expression level in the absence of inducer and; (iv) the inducer (arabinose) can be metabolised by wild-type *Salmonella*^31^.

Here, we have developed a new inducible lysis system based on the lambda phage lysis genes under transcriptional control of the tetracycline promoter/operator. Lysis is induced by Tetracycline or its analogue anhydrotetracycline (AHT), a low toxic antibiotic able to penetrate eukaryotic cells^32^. To produce the heteromacromolecule we have used a salicylate-inducible cascade expression system developed and optimised in our laboratory^33^,^34^, that is efficiently induced inside the eukaryotic cell in cultures and in animals *in vivo*^10^,^11^,^12^. This system combines a set of salicylate-regulated elements from *Pseudomonas putida* that works in cascade, containing a regulatory module (NahR and XylS2 transcription regulators coding sequences) integrated in the chromosome of *S. typhimurium sifA* mutant, and an expression module, consisting of a heterologous gene cloned under the control of the P_*m*_ promoter^33^. The *sifA* gene is necessary to maintain the integrity of the SCV and, therefore, bacteria carrying a mutation in this gene are released into the host cell cytosol several hours after internalisation^35^. After infection, the production of antitumoural molecules can be induced by salicylate and, when desired, delivery of the heteromolecule can be independently induced by AHT.

The p53 protein is a sequence-specific transcriptional factor that responds to different cellular stresses including DNA damage signals, oncogene expression, hypoxia or oxidative stress among others. *p53* is normally expressed at low levels in an inactive form that under stress conditions initiates a p53-dependent response leading to cell cycle arrest, senescence and/or apoptosis^36^. The *p53* gene is one of the most commonly mutated genes in human cancer. In some cases, *p53* mutations result in the loss of p53 protein expression. However, more frequently, *p53* tumour-associated alterations correspond to missense mutations that render p53 protein non-functional but stably expressed in tumours^37^. These mutations are frequently dominant negative over the wild type allele.

Previous studies have shown that the C-terminal p53 peptide (aa361–382) fused to the minimal carrier peptide *antennapedia* to facilitate peptide uptake from the growth medium, selectively induces cell death in malignant and pre-malignant cells with mutant or wild type p53 but is innocuous to normal cells^38^. This peptide has been described as selectively cytotoxic to breast, prostate, lung, glioma, mesothelioma and colon cancer cell lines in a p53-dependent manner^38^. This selectively cytotoxic peptide could open a new possibility for cancer treatment especially if it could be produced from inside the tumour, using an intratumourally accumulated bacteria.

Here, we have used a *Salmonella sifA*^*−*^ strain as an intracellular factory for the production of peptides in response to salicylate and their subsequent release through bacterial lysis triggered by AHT. To test the suitability of the system we have cloned and expressed the C-terminal p53 peptide. The results indicate that both, the expression and the lysis systems can be combined without disturbing bacterial growth or intracellular proliferation in the absence of inducer. Lysis induction causes the death of almost 100% of bacterial population, which releases the bacterial content into the growth medium or the host cytosol (if inside). Expressing Cp53 without the *antennapedia* domain and releasing it from the bacteria, resulted in clear cytotoxic effects. This result indicates that this induction and delivery system overcomes the requirement of fusing the cytotoxic peptide with signal peptides that could compromise the efficiency of treatment. In addition, our results revealed that temporal separation of protein production and protein release is essential to efficiently kill tumour cells.

## Results

### Construction of an AHT-inducible lysis system and characterisation of bacterial lysis in cultures

In order to develop an inducible lysis system to deliver molecules into the host cytosol, we have selected the bacteriophage lambda lysis gene cluster *SRRz*^39^. *S* encodes the holin that produces a collapse of the membrane potential and permeabilisation of the inner membrane^40^, *R* encodes the endolysin that degrades peptidoglycan, and *Rz* and *Rz1* are genes that encode the complex that spans the distance between the inner and outer membrane and disrupts the outer membrane^41^.

To construct a regulated lysis system, the *SRRz* cluster was cloned under the control of the *tet* promoter, which responds to tetracycline or tetracycline analogues such as AHT^42^. The tetracycline regulatory elements from plasmid pASK-IBA (IBA GmbH) were rearranged into the low copy vector pWSK29 and subsequently the *SRRz* cluster was cloned to obtain pMPO1632. The pWSK29 vector was selected for two reasons, first it has been demonstrated that this plasmid is stable during *Salmonella* infections^33^ and, second, the low copy number of the plasmid should reduce the potential toxicity of spurious expression of lysis genes. Plasmids bearing the lysis system were tested in *E. coli* DH5α and *Salmonella* strain MPO347 and lysis of the bacterial cultures was characterised. MPO347 strain bears the regulatory module of the salicylate inducible cascade expression system in its chromosome and also constitutively expresses the fluorescent dTomato protein^43^.

**Figure 1:**
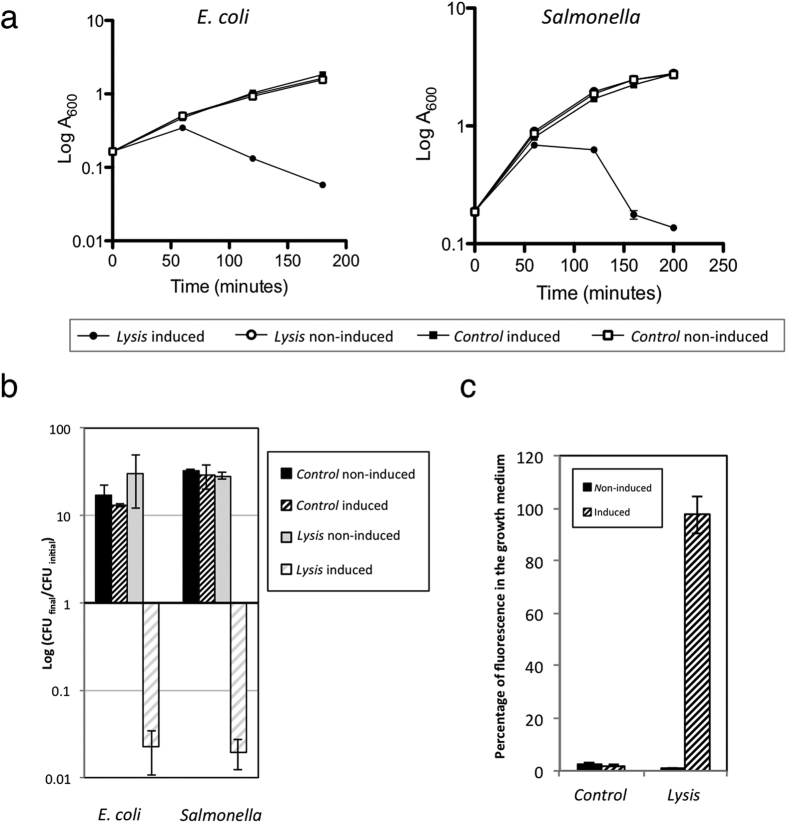
Bacterial lysis phenotype in bacterial cultures. (**a**) Monitoring of bacterial growth for bacterial cultures harbouring lysis (PMPO1632) or control plasmids (pMPO1631). Overnight cultures grown in LB, were diluted in fresh medium. When the A_600_ of the cultures reached 0.2 the cultures were divided in two groups and anhydrotetracicyle (AHT) was added to a final concentration of 0.2 μg/ml in half of the cultures (time 0). The optical density of induced and non-induced cultures was measured at regular time intervals. (**b**) Change in population size after induction. The number of viable cells was determined by cfu counting at time 0 and at the end of the experiment. The change in the population size was estimated as the ratio between the number of cfu at final time and the number of cfu at time 0. (**c**) The same samples of *Salmonella* were analysed for the presence of dTomato fluorescence in the growth medium. Graphics represents the mean ± SD of three independent experiments.

The results indicated that in absence of AHT, cultures continued proliferating independently of presence of the lysis system (Fig. 1a). However, the A_600_ of bacterial cultures carrying the lysis system decreased 1 h or 2 h after AHT induction for *E. coli* or *Salmonella*, respectively. The number of colony forming units (cfu) in LB plates decreased 100-fold when compared to the cfu at the time of induction and more than 1000-fold when compared to the non-induced strains or the induced control strain lacking the lysis system in both species (Fig. 1b). These results indicate that bacteria really die after lysis induction.

Subsequently, to verify that the lysis resulted in the release of the bacterial content into the culture medium, we quantified the fluorescence due to the cytosolic dTomato protein that was present in the supernatant of MPO347 cultures after lysis induction in *Salmonella*. As shown in Fig. 1c, almost 100% of the dTomato protein produced by bacteria was released into the growth medium after induction of bacterial cultures carrying the lysis system in contrast to non-induced and control strains in which the amount of released proteins was negligible.

Taken together, these results demonstrate that the developed lysis system does not compromise the viability of strains in the absence of inducer and that, in spite of the low copy number of the lysis cluster, most of bacterial population dies after induction and releases the bacterial content into the culture media.

### Salicylate induced protein production and lysis in bacterial culture

**Figure 2:**
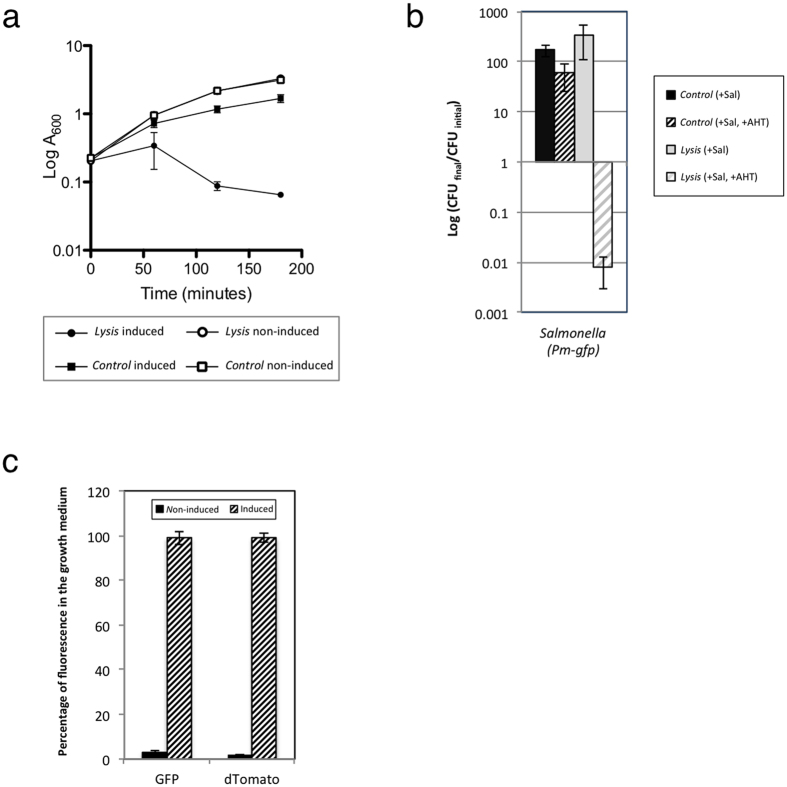
Combination of production and lysis systems in bacterial cultures. (**a**) Monitoring of bacterial growth for bacterial cultures harbouring lysis (pMPO1632) or control plasmids (pMPO1631) and the plasmid pMPO1604 for the production of GFP in response to salicylate. Overnight cultures grown in LB, were diluted in fresh medium. When the A_600_ of the cultures reached 0.2 the cultures were divided in two groups, salicylate was added in both groups whereas AHT was added in half of the cultures (time 0). The optical density of induced and non-induced cultures was measured at regular time intervals. (**b**) Change in population size after induction. The number of viable cells was determined by cfu counting at time 0 and at the end of the experiment. The change in the population size was estimated as the ratio between the number of cfu at final time and the number of cfu at time 0. (**c**) The same samples of *Salmonella* were analysed for the presence of dTomato or GFP fluorescence in the growth medium. The percentage of fluorescence in the media was calculated as in Fig. 1. Graphics represents the mean ± SD of three independent experiments.

As mentioned above, in our system the production of therapeutic proteins is induced by salicylate while the lysis system responds to AHT. Both systems were cloned into compatible plasmids and the ability of bacteria to maintain plasmids and respond to both inducers was tested. To that end, we tested the ability to produce and release GFP of *Salmonella* strain MPO347 bearing the appropriated combination of plasmids (Fig. 2). After lysis induction the optical density of cultures was monitored. As shown in Fig. 2a, A_600_ of bacterial cultures carrying the lysis plasmids decreased after induction, while the growth of cultures carrying the empty vector was not substantially affected in the presence of both inducers. As before, the clearance of cultures correlated with an approximately 100-fold reduction in the cfu number in LB plates (Fig. 2b).

To verify GFP production and release to the culture medium we analysed the amount of GFP protein into the supernatant of induced and non-induced bacterial cultures 3 h after induction. As before, almost 100% of the fluorescence (salicylate-induced GFP or constitutively produced dTomato) was detected in the supernatant in the presence of lysis systems and inducers in contrast to non-induced cultures where only a minor proportion of fluorescence (below 3%) was present in the supernatant (Fig. 2c).

Taking together these results indicate that both, the expression module and the lysis systems are compatible and that most of the protein produced after salicylate induction is released from bacteria upon induction of the lysis system.

### GFP production and delivery into HeLa cells cytosol

Next, we tested the system during bacterial infection of HeLa cells. The *Salmonella* strain MPO347 and its *sifA* derivative mutant MPO387 carrying plasmids pMPO1604 and pMPO1632 (*GFP-lysis*) or pMPO1631 (*GFP-control*) were used to infect HeLa cells. Once the invasion was established (1 h after invasion), GFP expression was induced with salicylate for 5 h. This time is long enough for the bacteria to induce GFP production while proliferating inside HeLa cells, and for the *sifA*^*−*^ strain to lose the SCV^35^. Next, AHT was added to the cultures to induce bacterial lysis for 15 h.

**Figure 3:**
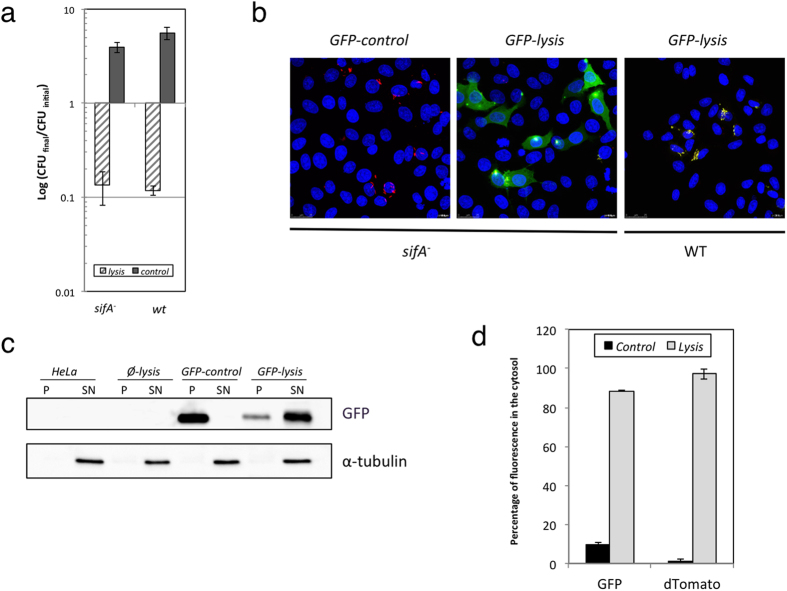
GFP production and delivery into HeLa cells citosol. HeLa cell cultures were infected with wt or *sifA*^*−*^ *Salmonella* bearing the different combinations of expression and lysis vectors. After 5 h of bacterial proliferation in the presence of salicylate in the culture medium, AHT was added (time 0) and cultures were analysed 15 h later. Graphics represents the mean ± SD of three independent experiments. (**a**) Change in intracellular population size after induction with AHT. The number of intracellular bacteria in both, wt and *sifA*^−^ strains, was determined by cfu counting at time 0 and at the end of the experiment. The change in the population size was estimated as the ratio between the number of cfu at final time and the number of cfu at time 0. (**b**) Immunostaining of GFP in HeLa cultures infected with *sifA*^*−*^ or *wt Salmonella* bearing the GFP expression vector and the control or lysis plasmids. (**c**) Western blot of GFP and α-tubulin of cytosolic (SN) and bacterial fractions (P) of HeLa cells cultures infected with *sifA*^*−*^ *Salmonella* bearing the empty (Ø) or the GFP (GFP) expression vector combined with control or lysis vectors. (**d**) Quantification of dTomato or GFP fluorescence in the host cell cytosol of HeLa cells infected with *Salmonella* bearing the GFP expression vector combined with control or lysis plasmids.

Results shown in Fig. 3a, indicate that the number of intracellular viable bacteria increased in both genetic backgrounds, wt or *sifA* mutant, when the strains bore the control plasmid. This increase in the number of bacterial counts corresponds to intracellular proliferation of bacteria during the infection. Conversely, when we compared the growth yield of bacteria carrying the lysis plasmid, both, wt and *sifA* mutant, exhibited a pronounced decrease in the viable bacteria compared to the initial intracellular population. In both cases, the viable intracellular bacteria decreased almost 5-fold with respect to the viable population previous to induction and 20 to 40-fold with respect to control strains. These results indicated that bacteria were dying intracellularly and, therefore, the lysis system functions properly when bacteria reside into host cells.

In order to detect bacterial content release into the host cytosol after lysis induction, we analysed by fluorescence microscopy HeLa cultures infected with wt and *sifA*^−^ *Salmonella* carrying the different combination of plasmids. Although GFP and dTomato fluorescence were easily detected previously to lysis induction, both disappeared several hours after lysis induction in cultures infected with bacteria carrying lysis plasmid (data not shown). This disappearance of intracellular bacteria did not correlate with fluorescence detection in the host cytosol. We speculated that after bacterial lysis, fluorescence was spread and diluted throughout the host cytosol so that the concentration of fluorescence was not sufficient for direct detection. In order to increase the sensitivity of the technique, we analysed cell cultures by immunostaining of GFP and fluorescence microscopy. During the immunostaining protocol, GFP lost its natural fluorescence while that of dTomato continued stable during the time course of the experiment. This allowed us to detect both proteins simultaneously by immunostaining just GFP. The results indicated that GFP produced by intracellular bacteria, after salicylate induction, was detected in the host cell cytosol only in *sifA*^*−*^ strains carrying the lysis plasmid (Fig. 3b, central panel), while no GFP (neither cytosolic nor intrabacterial) was detected in *sifA* mutant carrying the control plasmid (Fig. 3b, left panel). This was not due to lack of GFP production because GFP autofluorescence was easily detectable before the lysis and immunostaining treatment in all cases (data not shown). We deduce that, in the absence of lysis, bacteria were not permeable to the anti-GFP antibody making GFP undetectable. Conversely, lysis of wild type strain resulted in the detection of GFP associated to bacteria (Fig. 3b, right panel) indicating that, probably, bacteria were permeabilised but bacterial content remained enclosed inside the SCV.

Subcellular location of produced GFP was further demonstrated by Western blot analysis of cytosolic and bacteria together with cellular debris fractions. As shown in Fig. 3c, GFP protein was detected mainly in the cytoplasmic fraction only when bacteria carried the lysis plasmid. Conversely, GFP was absent in the supernatant of the control strain and was detected only in the pellet fractions (bacteria plus cellular debris). To quantify the percentage of bacterial protein released into the host cell, the *Salmonella* strain MPO387 carrying different combinations of plasmids (namely *GFP-lysis* and *GFP-control*), was used to infect HeLa cells. As shown in Fig. 3d, 90% of the GFP protein was located in the cytoplasmic fraction when *Salmonella* strain carried lysis plasmid while only a minor proportion (10%) of GFP was present into the host cytosol in HeLa cells infected with bacteria carrying the control plasmid. This small amount of cytoplasmic fluorescence in the absence of lysis may correspond to spontaneous death of intracellular bacteria. Similar results were obtained with dTomato fluorescence, which is produced constitutively by the bacteria. In this case, more that 95% of fluorescence was present into the host cytosol.

Therefore, these results indicate that the AHT-induced lysis system functions effectively during bacterial infection and, in combination with the *sifA* mutation, leads to the release of the produced protein into the host cell cytosol.

### Production and releasing of the proapoptotic peptide Cp53

To evaluate the suitability of our system as a tool against tumour cells, we tested the effect of Cp53 peptide intracellular production. As mentioned above, previous studies determined that Cp53 fused to the *antennapedia* domain induces apoptosis in malignant cells expressing wt or mutant p53^38^. The Cp53 peptide itself is able to rescue the transcriptional activation function of several p53 mutant proteins^44^ thus, the *antennapedia* domain is supposed to be required just for peptide internalisation. In our case, as the Cp53 peptide is produced intracellularly by the engineered bacterial strains, we anticipated that the *antennapedia* domain is not required to induce apoptosis.

MCF7 human breast cancer cell line harbours a wt p53 gene is sensitive to Cp53-*antennapedia*^38^. We infected MCF7 cells with *Salmonella* strains carrying combinations of the vector that produce Cp53 in response to salicylate (pMPO1649) or its empty vector control (pMPO52), and with the vector expressing the lysis system (pMPO1632) or its empty vector control (pMPO1631); in summary, we infected with four different *Salmonella* strains, namely *Cp53-lysis*, *Cp53-control*, *Ø-lysis* and *Ø-control*.

**Figure 4:**
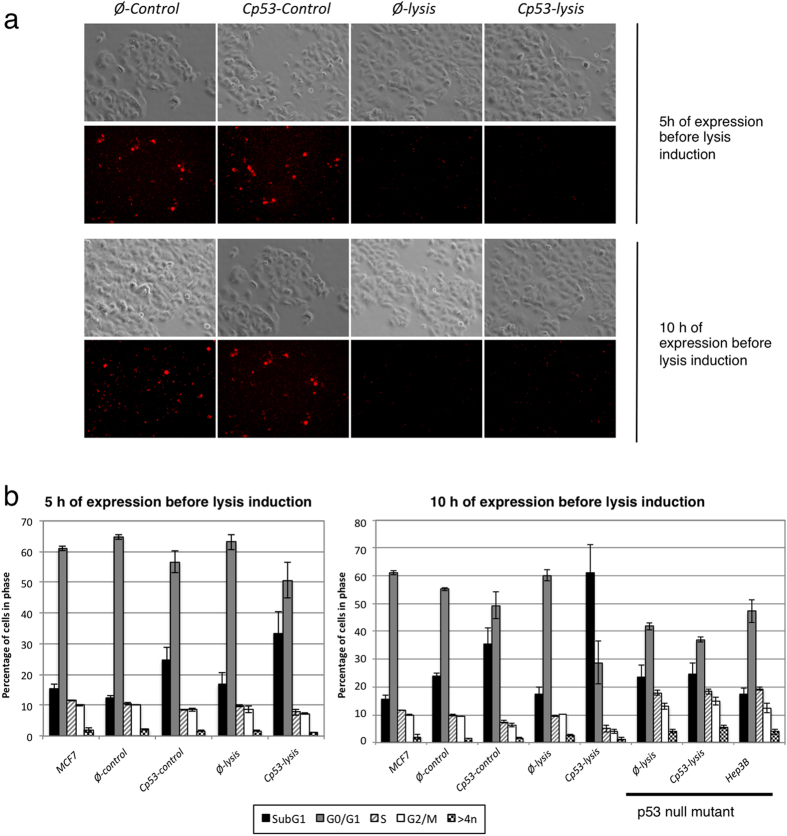
Cp53 production and delivery into host cells. MCF7 cells cultures were infected with *sifA*^*−*^ *Salmonella* (that express constitutively dTomato) bearing the different combinations of expression and lysis vectors. After 5 h or 10 h of bacterial proliferation in the presence of salicylate in the growth medium, AHT was added and cultures were analysed 15 or 10 h later. (**a**) Optical and fluorescence microscopy analysis of infected cultures at the end of the experiment. (**b**) Cell cycle analysis of control and infected MCF7 or Hep3B cultures at the end of the experiment. The SubG1 population correspond to apoptotic cells. Graphics represents mean ± SD of two independent experiments.

As above, 1 h after infection Cp53 expression was induced with salicylate and 5 h after Cp53 expression, lysis was induced with AHT for 15 h. To track the presence of *Salmonella* inside MCF7 cells, cultures were analysed by fluorescence microscopy at the moment of lysis induction and at the end of the experiment. Fluorescence microscopy analysis of MCF7 cultures indicated that lysis induction reduced significantly the final number of bacteria bearing the lysis vector (*Cp53-lysis* and *Ø-lysis*) but not in control strains (*Cp53-control* and *Ø-control*) (red dots in Fig. 4a), thus confirming that bacteria were efficiently lysed inside eukaryotic cells.

The effects of Cp53 production in these cell cultures were analysed by flow cytometry. Figure 4b shows the cell cycle distribution of MCF7 cell cultures. As usual, most of the cells (approximately 60%) of the non-infected control MCF7 cell cultures were in G0/G1 phase of the cell cycle. We expected that the expression and release of Cp53 produced an increase in the number of apoptotic cells (SubG1 population) and, concomitantly, a reduction of the G0/G1 population, as previously shown after induction of other apoptotic treatments^45^. As shown in Fig. 4b (left panel), production of Cp53 for 5 hours before lysis induction resulted in just a low increase (about 1.6 fold) in the SubG1 population of the MCF7 cells with a concomitant reduction of the G0/G1 population, indicating that production of Cp53 did not substantially increased the apoptotic population of cells.

We speculated that the time of Cp53 production before lysis induction could be insufficient to allow the accumulation of enough amount of the cytotoxic peptide to efficiently induce apoptosis, thus limiting the treatment efficiency. We therefore decided to allow Cp53 production for 10 h before lysis induction (Fig. 4b, right panel). In this condition, a very obvious difference, which inverted the relative abundance of SubG1 and G0/G1 in the cell cycle distribution, was observed when MCF7 cells were infected with *Cp53-lysis* bacteria: The percentage of SubG1 population increased to approximately 60% while G0/G1 decreased to 28%. In contrast, when the same experiment was carried out in the Hep3B cell line (null mutant of P53), despite having similar infection rate than before (~60–70%), the percentage of SubG1 population remained unaltered when cells were infected with *Cp53-lysis* bacteria. These results indicate that the apoptosis induced by the Cp53 peptide requires the presence of P53 protein in the host cell, as previously described^38^.

In summary, these results clearly show that inducing Cp53 production *(Cp53-lysis*) inside a *Salmonella sifA* mutant that is infecting a MCF7 tumour cell, and subsequent bacterial autolysis induction to release the produced peptide, is an efficient treatment to induce apoptosis of the tumour cell. However, production of the cytotoxic peptide by the bacteria and its lysis induction has to be sufficiently separated in time to allow enough accumulation of the cytotoxic peptide to exert its effect when released into the eukaryotic cytosol.

## Discussion

Over the past decade, many genera of bacteria have been explored as cell factories for cancer therapy due to their ability to specifically target tumours^2^. *Salmonella typhimurium* is probably the intracellular pathogen that has been most extensively studied as an anti-tumour vector. The natural ability of *Salmonella* to proliferate inside tumours and to induce tumour regression can be upgraded by modifying *Salmonella* to produce therapeutic agents, thus making the bacteria an optimised machine for cancer therapy. However, numerous challenges remain before that perfect intratumoural bacterial factory could be developed. Among them, the accurate timing of therapeutic production and releasing are important limitations. Inappropriate production of therapeutic proteins during infection could compromise bacterial capacity to grow, to avoid the immune system and to reach the intratumoural environment, thus limiting the treatment efficiency and specificity. For these reasons, the use of an expression system finely regulated that trigger therapeutics production in response to an inducer safe for human use that have a good bio-distribution, is important to ensure the safety and the success of the treatment. In previous studies we have developed and optimised a cascade expression system that is tightly regulated and induced by salicylate or acetyl-salicylic acid (ASA)^10^,^11^,^12^,^33^,^34^.

Another important constraint is the efficient release of the therapeutic agents. Most studies have been focused on therapeutic production, ignoring that most of the antitumoural agents produced would remain inside the bacteria or the SCV, thus limiting the treatment efficiency. Using a controlled autolysis system to release bacterial content, any kind of macromolecule produced by bacteria could be delivered into the tumour site. However, this requires controlled expression of the lysis genes so that the system is turned off until bacteria accumulate in the tumour and produce enough therapeutic to be efficient.

In this work we have improved *Salmonella* for efficient delivery of therapeutic agents. For this purpose, we have constructed an inducible autolysis system based on the lambda phage that allows the releasing of therapeutic agents into the extracellular medium or the eukaryotic cytosol, and combined it with a second inducible system responsible for producing heterologous proteins to high levels^33^. Both regulatory systems respond to different inducer molecules, AHT (or other tetracycline analogues) in the case of the autolysis, and salicylate or ASA (aspirin) in the case of the heterologous protein production, which allows inducing each system independently. Additionally, both inducers could eventually be administered to patients, since its use in humans is approved. Tetracycline and its analogues (doxytetracycline or anhydrotetracycline) are good inducer molecules as they regulate gene expression at very low concentrations, can penetrate both bacterial and animal cells, are non-toxic at the necessary levels and can be used *in vivo*^10^,^32^,^46^. On other hand, ASA is one of the most widely used and best-characterised analgesics^47^,^48^. Additionally, the use of *sifA* mutants allows surpassing the SCV barrier without expressing any additional protein, such as listeriolysine used in other systems^24^,^25^,^26^,^27^, simplifying this way the delivery system.

Neither bacterial growth nor bacterial infection, were disturbed by the lysis plasmid, indicating that lysis is conveniently turned off. However, lysis induction caused the death of almost the totality of the bacterial population, and the release of the bacterial content into the growth medium or the host cytosol. This solves an additional concern regarding bacterial therapy that is the clearance of bacteria after treatment. Using this autolysis system we kill most part of bacterial population, thus increasing the safety of bacterial therapy. The surviving bacteria, that should be the vast minority, could be easily cleared using antibiotics after treatment.

The coexistence of both expression systems (the production and the lysis systems) without compromising the efficiency of each other is crucial for a successful anti-tumour treatment since production of the therapeutic peptide by salicylate induction could not be subsequently switch off before inducing bacterial autolysis *in vivo*. Our results demonstrate that, in bacterial cultures, both systems can be simultaneously induced. In this case, the time elapsed between lysis induction and effective bacterial death was long enough to detect GFP production in response to salicylate and almost 100% of the GFP protein produced was released into the growth medium as the result of cell lysis.

However, as production and lysis respond to different inducers, it is possible to separate production of therapeutic agent from its subsequent release to allow sufficient production prior to delivery. This property is particularly relevant since this allows production of therapeutics molecules while *Salmonella sifA* mutant proliferates and is being released into the host cell cytosol. Considering that release of a *Salmonella sifA* strain into the host cell cytosol requires several hours from its infection in cultured cells^35^, we decided to induce protein production at the time of infection and lysis 5 hours after infection, when the bacteria are no longer surrounded by SCV. During this time salicylate was present into the growth medium so that bacteria could produce the desired protein while they lose the SCV. Using this strategy to produce and deliver GFP intracellularly, bacteria released 90% of the GFP into the host cytosol, which represented an amount of protein enough to stain the host cell cytosol. Conversely, just a minor proportion (10%) of protein was released in the control strains. This proportion, which corresponds to the amount of protein released by spontaneous bacterial death, confirms that, in the absence of lysis, most of the therapeutic molecules remain useless inside the bacteria, thus limiting the treatment efficiency. Interestingly, the lysis system destroys the first barrier (bacterial envelope) but it is not sufficient to release the produced protein into the host cytoplasm, thus indicating that protein release outside the bacteria is not enough to efficiently deliver the protein due to the additional SCV barrier (Fig. 3c). Nevertheless, the use of a *sifA* mutant (Fig. 3b) allows direct release of the bacterial content into the host cytosol upon lysis induction because the SVC disappears^35^.

Cp53 peptide fused to *antennapedia* domain has been proposed as a novel agent with unique selectivity for human cancer cells. The main limitation of this molecule in cancer treatment is how to bring the peptide inside the tumour. Using the engineered bacteria, we could produce this peptide directly in the tumour environment facilitating its access to the tumour cell cytosol. Interestingly, our results showed that sufficient separation in time of protein production from the subsequent lysis is essential for efficient killing of tumour cell. This confirms that, depending on the amount of therapeutic molecule required, the system in which lysis and production respond to the same inducer could be inefficient because the bacteria may die before the cytotoxic peptide is produced to levels sufficient to kill the host cell.

Using the combined system, bacteria were able to induce the death of more than 60% of tumour cell population. This effect must be even higher considering that not all MCF7 cells were infected by *Salmonella* and exposed to Cp53. Although the experimental conditions are not comparable it is noteworthy that, in previous studies, MCF7 cells treated with 30 μM of purified C53p-Ant for 6 h, induces apoptosis in 30–40% of cell population^38^,^49^. In our case, with a percentage of infection ranging between 60–70% of the cell culture, we observed an increase in the apoptotic population up to more than 60% when peptide production is allowed for 10 h previous to releasing. This indicates that the *in situ* production system may be even more efficient than an external treatment with a high amount of purified peptide. Additionally, the system is useful to release peptides intracellularly avoiding the need of fusions with cell-penetrating peptides that could compromise the efficiency of the treatment and restrict the nature of the therapeutic agents.

The transition from general cytotoxic chemotherapy to specific molecules against tumour cells is expected to create more effective and selective anticancer treatments in the future. In this sense, an increasing number of new molecules potentially active against cancer are continuously emerging^27^,^50^,^51^,^52^,^53^,^54^,^55^. The main limitation for testing these molecules, i. e. how to bring these molecules inside the tumour, can be clearly solved by the use of engineered *Salmonella*. Our combined system for intracellular drug production and release into the host cytosol could allow easy testing in cell cultures therapeutic molecules against cancer. Those molecules showing an efficient anticancer activity could also be produced and delivered *in vivo* by the engineered bacteria. The results presented here indicate that our engineered *Salmonella* is a powerful tool for the production and delivering of new anticancer molecules, opening the door to new anticancer treatments.

## Methods

### Bacterial strains and growth conditions

Bacterial strains used in this work are described in Supplementary Table S1. Cultures were grown aerobically at 180 r.p.m. and 37 °C in Luria-Bertani (LB) medium and supplemented with antibiotics when necessary. Transductional crosses using phage P22 HT 105/1 int201^56^ were used for transferring chromosomal markers among the strains.

### Molecular biology general procedures

All DNA manipulations were performed following standard protocols^57^. All oligonucleotides used in this study are described in Supplementary Table S2.

GFP gen from pFV25-1 (fragment XbaI-HindIII)^16^ was cloned under the P_*m*_promoter control into pMPO51 plasmid^33^, thus generating plasmid pMPO1604.

The Cp53 fragment under the P_*m*_ promoter control was constructed by cloning two annealed complementary oligonucleotides Cp53-Fw and Cp53-Rev, digested with NdeI-HindIII into pMPO52 NdeI-HindIII, thus generating plasmid pMPO1649.

pWSK29 plasmid was modified to introduce regulatory elements from pASKIBA43 and the lysis cluster. Briefly, the *tetR* gen from pASKIBA43 was cloned under the P_*Bla*_ promoter for constitutive expression of TetR repressor. The *SRRz* (1917 pb) cluster was obtained by PCR amplification of lambda DNA using primers ADN lisFw and lislabdrev and cloned under the P_*tet*_ promoter (from pASKIBA43) upstream and in opposite direction to *tetR* gen. The chloramphenicol resistance gen from pKD3, was subsequently cloned upstream *tetR* to generate plasmid pMPO1086. Finally, the ampicillin resistance originally present in pWSK29 was eliminated by digestion of pMPO1086 with PvuI, thus generating plasmid pMPO1632. Control plasmid pMPO1631 contains all the above elements but lacks SRRz. Supplementary Fig. S1 shows the most important features of both expression and lysis plasmids.

### Construction of *Salmonella* strains

To integrate DNA sequences into the *Salmonella* chromosome we used a modification of the method published by Datsenko and Wanner^58^, as described in^10^. Kanamycin or chloramphenicol resistance genes were cloned into plasmids bearing the sequences to be integrated at the chromosome. Using this strategy we generated strain MPO340 by replacing the sequence P_*taq*_-*gfp* KmR from MPO96^33^ with P_*taq*_-*dtomato* CmR (plasmid pMPO1065). Subsequently, the antibiotic resistance of the regulatory module was excised using pCP20 plasmid and verifying by PCR using primers xylSFw2 and tomatoHindIIIR generating finally MPO347 strain.

For the construction of the *sifA* mutant strain we amplified the kanamycin resistance gene from pKD4 with primers antifinsifA-P1 and prinsifa-P2. After the generation of primary mutants (MPO38), the excision of the Kanamycin resistance was induced using pCP20 plasmid and tested by PCR amplification using primers sifAE1 and Sac-P1, thus generating strain MPO42. Next, the regulatory module from MPO340 (see above) was transduced by P22 phage into MPO42 strain, thus generating MPO386 strain. Subsequently, the antibiotic resistance of the regulatory module was excised using pCP20 plasmid and verifying by PCR using primers xylSFw2 and tomatoHindIIIR to generate the final MPO387 strain.

### Lysis induction in bacterial cultures

Bacteria bearing the specific combination of plasmids were grown at 37 °C in LB supplemented with the appropriate antibiotics. Overnight cultures were subsequently diluted in LB and, once the optical density (A_600_) reached 0.2, each culture was divided in two groups. One of the duplicated groups was induced with AHT to a final concentration of 0.2 μg/ml (the induced group). The other set of cultures were the non-induced group. When necessary, both groups were induced with salicylate 2 mM. Both sets of cultures were incubated at 37 °C for the time specified in each experiment. The A_600_ of induced and non-induced cultures was measured at regular time intervals. Dilutions of cultures were spread in LB plates without antibiotic to determine the number of viable bacteria by colony-forming units (cfu) counting.

### *In vitro* bacterial infection of tumour cells

Tumour cells infections were performed as described elsewhere^33^ with minimal modifications. Cells were cultured in 24-well plates at a density of 10^5^ cells per well, or in 6-well plates at a density of 2 × 10^5^ cells per well, 20 h before infection. An overnight *Salmonella* culture was diluted 1:33 on fresh LB supplemented with ampicillin (100 μg/ml) and chloramphenicol (15 μg/ml) when necessary, and incubated at 37 °C during 3.5 h. For infection, bacteria were added at multiplicity of infection (m.o.i.) of 100:1 or 250:1 allowing the infection to proceed for 20 min at 37 °C and 5% CO2. Wells were washed twice with Phosphate Buffer Saline (PBS) and incubated for 1 h with Dulbecco’s modified Eagle’s medium (DMEM; Sigma) containing 100 μg/ml gentamicin (PAA laboratories GmbH, Austria) to kill extracellular bacteria. After that, the antibiotic concentration was reduced to 16 μg/ml, and 2 mM sodium salicylate (Sigma-Aldrich, Germany) was added to the culture medium to induce protein expression. After 5 or 10 h of salicylate induction, 0,2 μg/ml of AHT (Clontech Laboratories) was added and lysis expression was allowed for 13–18 h until analysis.

For counting of intracellular bacteria (gentamicin-protected), eukaryotic cells were washed three times with PBS, gently lysed with 0.1% Triton X-100 for 10 min^35^, and dilution series were plated onto LB agar. HeLa cells but not the bacteria are broken by this treatment.

### Western blot analysis

HeLa cells infected with MPO387 bearing the appropriate combination of plasmids at a m.o.i of 250:1 (see above), were induced with salicylate for GFP production for 5 h and subsequently 15 h with AHT. Subcellular fractioning was performed as described previously^33^. Cells were detached by trypsin treatment washed twice with cold PBS and resuspended on 200 μl of Lysis Buffer^59^ and kept on ice for 30 min. The cell lysate was centrifuged at 13,000 r.p.m. for 10 min., the supernatant (cytoplasmic fraction) was retired and stored, whereas the pellet (bacteria and cellular debris) was washed twice with PBS and finally resuspended in the original volume of Lysis buffer (200 μl). Protein concentrations were determined by the QuantiPro^TM^ BCA assay (Sigma-Aldrich) and 30 μg of protein from the pellet and an equivalent volume of supernatant were boiled in 2XSDS sample buffer and loaded on SDS-PAGE. After electrophoresis and Western blotting, immunoreactive products to anti-GFP, and anti-α-tubulin (DM1A, Calbiochem, 1:7500) were detected as above.

### GFP and dTomato quantification

#### Bacterial cultures

*Salmonella* cultures were induced for production and lysis as mentioned above. Aliquots of cultures taken at 0 and 3 h post induction were passed through a 0.22 μm filter.

Cell density and GFP (excitation 485BP1 and emission EM520 filters, Gain 1000) and dTomato (excitation 540/10 and emission 620/10 filters, Gain 2000) fluorescence of non-filtered and filtered cultures were measured with a Polar Star Omega microtitre plate fluorometer (BMG LABTECH, Germany). The fluorescence values were corrected for the background fluorescence. The percentage of fluorescence in the media was calculated as (filtered culture fluorescence/non-filtered culture fluorescence) ×100.

#### Infected eukaryotic cells

Subcellular fractioning of 5 × 10^5^ HeLa cells, that were previously infected with MPO387 bearing the appropriate combination of plasmids at a m.o.i. 250:1, were carried out as above. Detached by trypsin treatment washed twice with cold PBS and then resuspended on 450 μl of Lysis Buffer and kept on ice for 30 min. The cell lysate was centrifuged at 13,000 r.p.m. for 10 min, the supernatant (cytoplasmatic fraction) was taken and stored, whereas the pellet (bacteria and cellular debris) was washed twice with PBS and finally resuspended in the original volume of lysis buffer (450 μl). GFP and dTomato fluorescence were measured as above. The fluorescence values were corrected for the background (non infected HeLa cells). The percentage of fluorescence in the cytosol was calculated as (supernatant fluorescence/total fluorescence) ×100.

### Fluorescence immunostaining

For fluorescence staining of GFP, infected cells were grown on glass coverslips (12 mm, Thermo Scientific) and GFP production and bacterial lysis was induced as mentioned before. Cell samples were taken 15 h after lysis induction and rinsed twice with PBS, fixed in 3.7% paraformaldehyde for 10 min at room temperature and permeabilised in 0.1% Triton X-100 for 10 min. Thereafter, cells were washed twice with PBS and incubated for 90 min with 1:250 anti-GFP (A6455- invitrogen) at 37 °C. Cells were incubated for 90 min with the ALEXA FLUOR 488 goat anti-rabbit (A11008- life technologies) secondary antibody at 37 °C. Subsequently, cells were washed twice with PBS and incubated for 15 min with PBS containing Hoechst 33258 (1 μg/ml) at room temperature in the dark. After washing with PBS the coverslips were mounted on slides and visualised with a confocal microscope Leica SPE (630X) (Leica Microsystems GmbH, Wetzlar, Germany).

### Cell cycle analysis

2 × 10^5^ MCF7 or Hep3B cells were previously infected with MPO387 bearing the appropriate combination of plasmids at m.o.i 100:1, as described above. Cell distribution was determined by flow cytometry of propidium iodide (PI)-stained nuclei^12^. The harvested cells (approx. 5 × 10^5^ cells) were washed twice with PBS and fixed in 80% cold ethanol at −20 °C for at least 24 h. After fixation, cells were washed twice with PBS containing 0.1% BSA and the pellets were resuspended in phosphate-citrate buffer (0.2 M Na2HPO4, 0.1 M citric acid pH 7.8) for 5 minutes at RT. After centrifugation at 1800 r.p.m for 5 min, the cell pellet was resuspended in DNA staining solution (100 μg/ml of RNase A (R5125; Sigma- Aldrich), 40 μg/ml of PI, 0.1 mM EDTA pH 8 and 0.1% Triton X-100, in PBS) and incubated 30 min at 37 °C in the dark. Samples were analysed by flow cytometry. 10,000 events for each sample were analysed using CellQuest software to determine the relative DNA content based and to evaluate the percentages of cells in sub-G1 (apoptotic cells), G0/G1, S, and G2/M phases. Results were presented as mean ±SD.

## Additional Information

**How to cite this article**: Camacho, E. M. *et al*. Engineering *Salmonella* as intracellular factory for effective killing of tumour cells. *Sci. Rep.* **6**, 30591; doi: 10.1038/srep30591 (2016).

## Supplementary Material

Supplementary Information
