# Author Correction: Engineering *Salmonella* as intracellular factory for effective killing of tumour cells

**DOI:** 10.1038/s41598-026-51992-z

**Published:** 2026-06-04

**Authors:** Eva María Camacho, Beatriz Mesa-Pereira, Carlos Medina, Amando Flores, Eduardo Santero

**Affiliations:** Centro Andaluz de Biología del Desarrollo/CSIC/Universidad Pablo de Olavide/Junta de Andalucía. Departamento de Biología Molecular e Ingeniería Bioquímica, Seville, Spain

Correction to: *Scientific Reports* https://doi.org/10.1038/srep30591, published online 28 July 2016

The Article contains errors in Fig. 4.

As a result of errors during figure assembly, in the 5 h panels,

- both images in the ϕ-control group were duplicates of the corresponding images from the Cp53-Control group in the 10 h panels
- both images in the ϕ-lysis group were duplicates of the corresponding images from the Cp53-lysis group in the 10 h panels

The correct Figure 4 appears below.

**Fig. 4:**
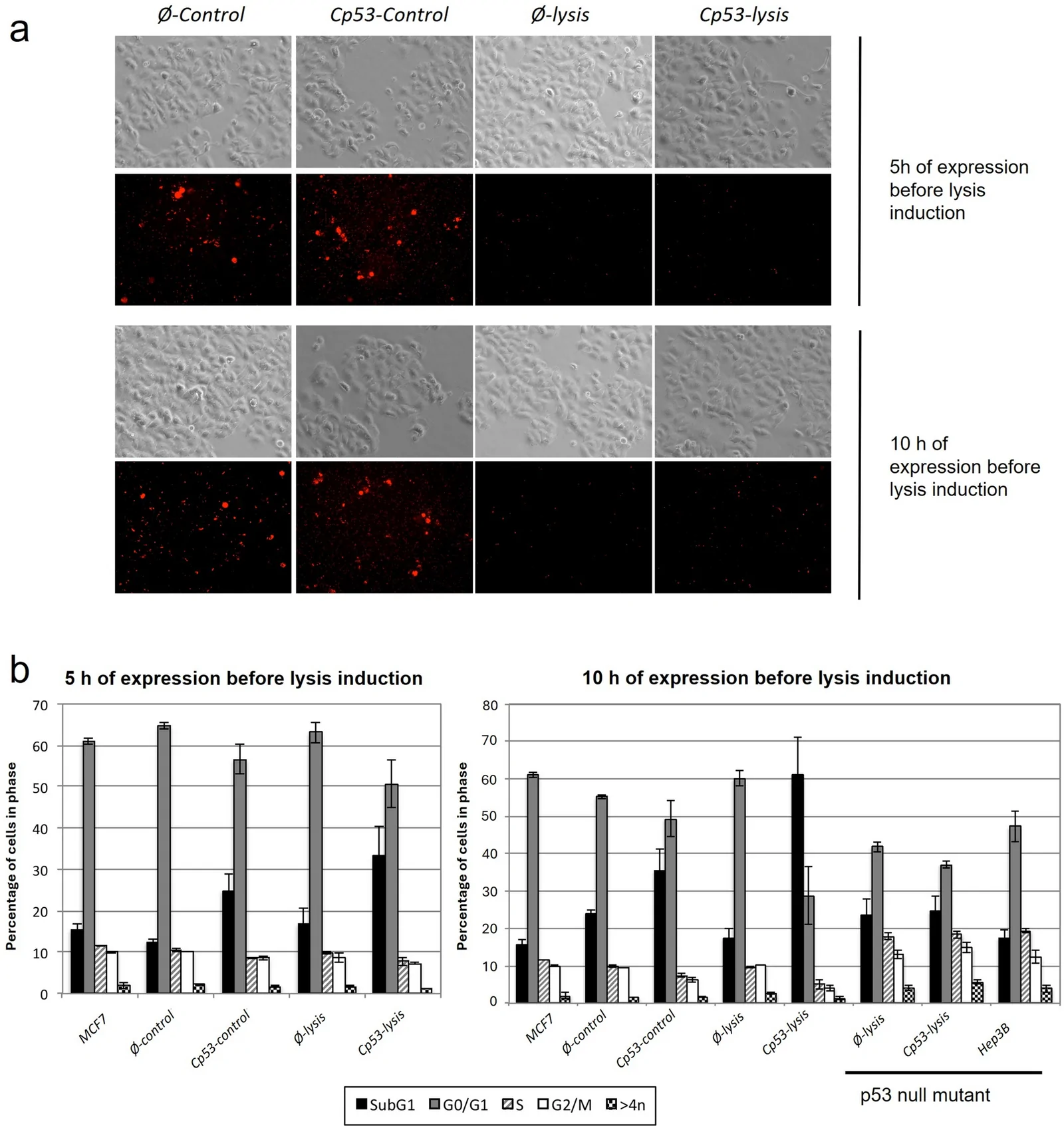
Cp53 production and delivery into host cells. MCF7 cells cultures were infected with *sifA*^*−*^ Salmonella (that express constitutively dTomato) bearing the different combinations of expression and lysis vectors. After 5 h or 10 h of bacterial proliferation in the presence of salicylate in the growth medium, AHT was added and cultures were analysed 15 or 10 h later. (**a**) Optical and fluorescence microscopy analysis of infected cultures at the end of the experiment. (**b**) Cell cycle analysis of control and infected MCF7 or Hep3B cultures at the end of the experiment. The SubG1 population correspond to apoptotic cells. Graphics represents mean ± SD of two independent experiments.

