# Supplementary material for: Engineering Salmonella as intracellular factory for effective killing of tumour cells
Source: Sci Rep. 2016 Jul 28;6:30591. doi: 10.1038/srep30591 (PMC4964584; doi:10.1038/srep30591)
Supplement: Supplementary Information [file srep30591-s1.pdf]

Engineering *Salmonella* as intracellular factory for effective killing of tumour cells

Eva María Camacho*, Beatriz Mesa-Pereira, Carlos Medina, Amando Flores, Eduardo Santero.

Centro Andaluz de Biología del Desarrollo/ CSIC/ Universidad Pablo de Olavide/ Junta de Andalucía. Departamento de Biología Molecular e Ingeniería Bioquímica, Seville, Spain

Supplementary Figure S1:

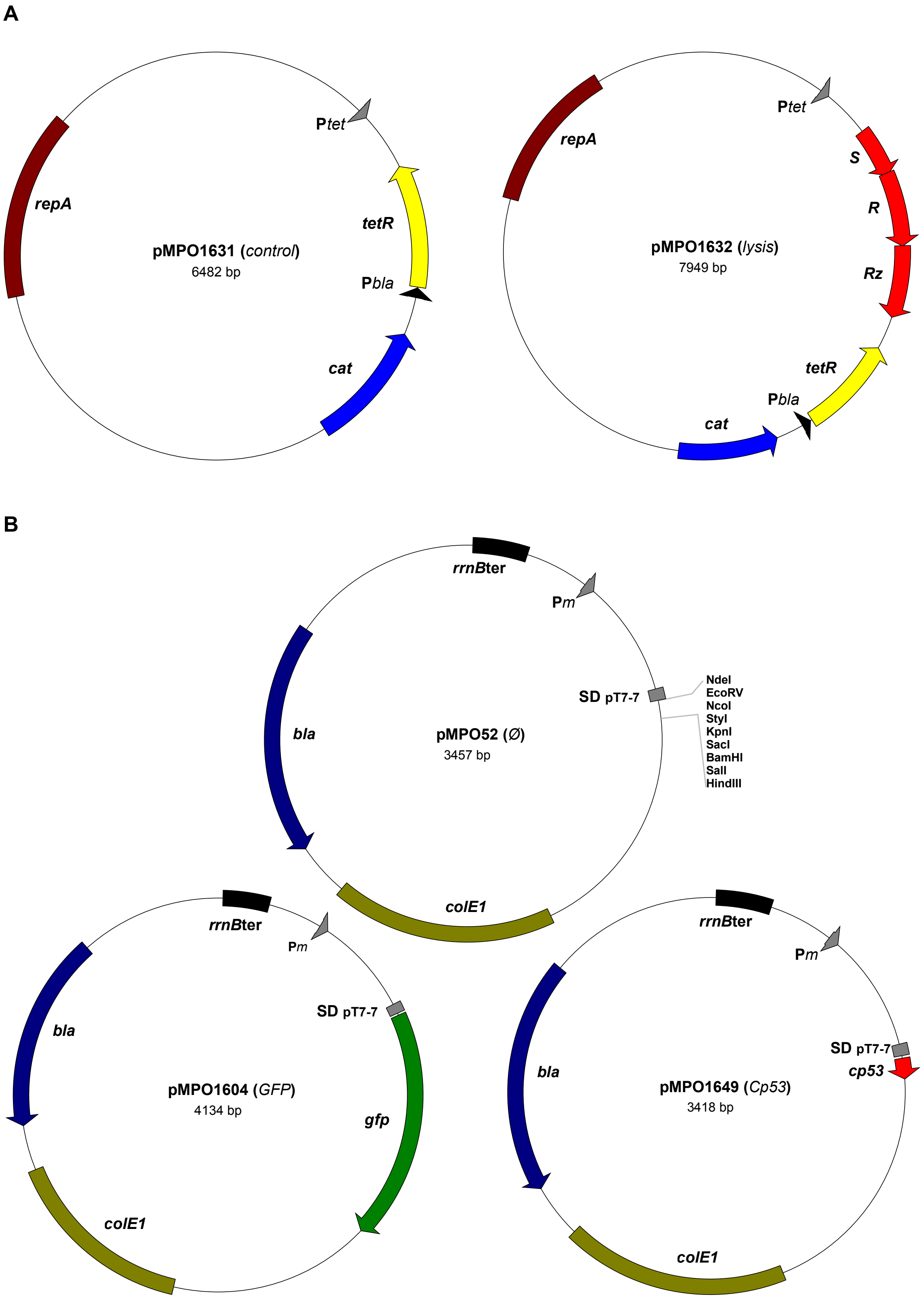

Figure S1: Schematic diagram of the lysis and expression plasmids used in this study. (A) Low copy number plasmid (pSC101 replicon) containing the lambda lysis cluster (pMPO1632 or Lysis) and its control (pMPO1631 or control). (B) Vector for GFP (pMPO1604 or gfp) and Cp53 (pMPO1649 or Cp53) expression and the empty vector pMPO52

Supplementary Table S1. Strain and plasmids

	Characteristics	Reference
***E. coli* strains**		
DH5α	*deoR endA1 gyrA96 recA1 supE44*	[1]
***S. typhimurium* strains**		
14028	Wild type strain	[2]
MPO38	Δ*sifA::Km*	This work
MPO42	Δ*sifA*	This work
MPO96	14028 Δ*trg:: (nahR/*P_{sal}*–xylS2–nasR*/Km/P_{Tac}*–gfp)* fusion	[3]
MPO340	14028 Δ*trg:: (nahR/*P_{sal}*–xylS2–nasR*/Cm/P_{Tac} *–dTomato)* fusion	This work
MPO386	14028 Δ*sifA* Δ*trg:: (nahR/*P_{sal}*–xylS2–nasR*/Cm/P_{Tac} *–dTomato)* fusion	This work
MPO387	14028 Δ*sifA* Δ*trg:: (nahR/*P_{sal}*–xylS2–nasR*/P_{Tac} *–dTomato)* fusion	This work
**Plasmids**		
pASKIBA43plus	ApR, expression plasmid. The expression cassette is under transcriptional control of the tetracycline promoter/operator	IBA Biotag technology
pCP20	ApR, CmR, Ts (30ºC)	[4]
pFPV25-1	*rpsM::gfp* mut	[5]
pKD3	ApR, CmR, OriRγ	[6]
pKD4	ApR, KmR, OriRγ	[6]
pKD46	ApR, *oriR101*, *repA101*(ts), *araBp-gam-bet-exo*	[6]
pMPO51	ApR, expression vector with rrnBT1T2-P_m-MCSII, ColE1 replication origin	[3]
pMPO52	ApR, expression vector with rrnBT1T2-P_m-T7 SD sequence-MCSII, ColE1 replication origin	[3]
pMPO1065	Derived from pMM40 [7]. ApR, CmR-His t-P_{Tac}*–tdTomato (Clontech Laboratories)*	This work
pMPO1086	ApR, CmR, pWSK29 derived plasmid with P_m*-SRRz* and P_{bla}*-tetR*	This work
pMPO1604	ApR, pMPO51 derived plasmid with *gfp* cloned downstream Pm	This work

pMPO1631	CmR, pWSK29 derived plasmid with P_{bla}-*tetR*	This work
pMPO1632	pMPO1086 derived plasmid with CmR, P_{m}-*SRRz* and P_{bla}-*tetR*	This work
pMPO1649	ApR, pMPO52 derived plasmid with *cp53* cloned downstream Pm	This work
pWSK29	ApR cloning vector	[8]

REFERENCES

1 Hanahan, D. Studies on transformation of *Escherichia coli* with plasmids. *J. Mol. Biol.* **166**, 557-580 (1983).

2 Fields, P. I., Swanson, R. V., Haidaris, C. G. & Heffron, F. Mutants of *Salmonella typhimurium* that cannot survive within the macrophage are avirulent. *Proc. Natl. Acad. Sci. U S A* **83**, 5189-5193 (1986).

3 Medina, C., Camacho, E. M., Flores, A., Mesa-Pereira, B. & Santero, E. Improved expression systems for regulated expression in *Salmonella* infecting eukaryotic cells. *PloS one* **6**, e23055, doi:10.1371/journal.pone.0023055 (2011).

4 Cherepanov, P. P. & Wackernagel, W. Gene disruption in *Escherichia coli*: TcR and KmR cassettes with the option of Flp-catalyzed excision of the antibiotic-resistance determinant. *Gene* **158**, 9-14, doi:037811199500193A [pii] (1995).

5 Valdivia, R. H., Hromockyj, A. E., Monack, D., Ramakrishnan, L. & Falkow, S. Applications for green fluorescent protein (GFP) in the study of host-pathogen interactions. *Gene* **173**, 47-52 (1996).

6 Datsenko, K. A. & Wanner, B. L. One-step inactivation of chromosomal genes in *Escherichia coli* K-12 using PCR products. *Proc. Natl. Acad. Sci. U S A* **97**, 6640-6645, doi:10.1073/pnas.120163297 (2000).

7 Kleiner, D., Paul, W. & Merrick, M. J. Construction of multicopy expression vectors for regulated over-production of proteins in *Klebsiella pneumoniae* and other enteric bacteria. *J. Gen. Microbiol.* **134**, 1779-1784, doi:10.1099/00221287-134-7-1779 (1988).

8 Wang, R. F. & Kushner, S. R. Construction of versatile low-copy-number vectors for cloning, sequencing and gene expression in *Escherichia coli*. *Gene* **100**, 195-199 (1991).

Supplementary Table S2. Primers used for this work

Primer name	Sequence (5'->3')
antifinsifA-P1	aaaacctgatcagagatggcttcgactatctcagctaacatactttggcttgtaggctggagctgcttc
Cp53-Fw	tatgcgcatatggggagcagggctcactccagccacctgaagtccaaaaagggtcagtctacctcccgccattaagctttat
Cp53-Rev	ataaagcttaatggcgggaggtagactgacccttttggacttcaggtggctggagtgagccctgctccccatatgcgcata
lisFw	acggatggcaacatattaac
lislabdrev	atatgggcaactctatctgc
prinsifa-P2	tctacatgagatgttgtttgccgaacgcgcccccacacgagagcggcttacatatgaatatcctccttag
Sac-P1	tatagagctctgtaggctggagctgcttc
sifAE1	tactccagtataagtgag
tomatoHindIIIR	taaagcttttacttgtacagctcgtcc
xylSFw2	tgaacgatcccagtgccaatgtgc
